# Sub-national mapping of population pyramids and dependency ratios in Africa and Asia

**DOI:** 10.1038/sdata.2017.89

**Published:** 2017-07-19

**Authors:** Carla Pezzulo, Graeme M. Hornby, Alessandro Sorichetta, Andrea E. Gaughan, Catherine Linard, Tomas J. Bird, David Kerr, Christopher T. Lloyd, Andrew J. Tatem

**Affiliations:** 1 WorldPop, Department of Geography and Environment, University of Southampton, Southampton SO17 1BJ, UK; 2 Flowminder Foundation, Roslagsgatan 17, Stockholm SE-11355, Sweden; 3 GeoData, University of Southampton, Southampton SO17 1BJ, UK; 4 Department of Geography and Geosciences, University of Louisville, Louisville, Kentucky 40292, USA; 5 Department of Geography, University of Namur, Namur B-5000, Belgium; 6 Spatial Epidemiology Lab (SpELL), Université Libre de Bruxelles, Brussels B-1050, Belgium

**Keywords:** Environmental social sciences, Developing world, Geography

## Abstract

The age group composition of populations varies substantially across continents and within countries, and is linked to levels of development, health status and poverty. The subnational variability in the shape of the population pyramid as well as the respective dependency ratio are reflective of the different levels of development of a country and are drivers for a country’s economic prospects and health burdens. Whether measured as the ratio between those of working age and those young and old who are dependent upon them, or through separate young and old-age metrics, dependency ratios are often highly heterogeneous between and within countries. Assessments of subnational dependency ratio and age structure patterns have been undertaken for specific countries and across high income regions, but to a lesser extent across the low income regions. In the framework of the WorldPop Project, through the assembly of over 100 million records across 6,389 subnational administrative units, subnational dependency ratio and high resolution gridded age/sex group datasets were produced for 87 countries in Africa and Asia.

## Background & Summary

Populations in low income countries are generally younger and growing more rapidly compared to those in high income countries. Africa has the highest rate of population growth and more than half of the global population growth between now and 2050 is expected to occur in Africa^1^. Asia is projected to be the second largest contributor to future global population growth, adding 0.9 billion people between 2015 and 2050^1^.

Population demographics have been central to the Millennium Development Goals and are key to the Post-2015 Sustainable Development Agenda^2^, and development policies need to account for population dynamics and their relationships with social, economic and environmental factors^3,4^. One aspect characterising low income countries is the higher proportion of children relative to working age individuals^5,6^. Moreover, given the increasing number of elders dependent on working age individuals, population aging is also an emerging issue in some low and middle income countries^7,8^. The ratio between the share of the ‘dependent age’ population (either <15 years or >65 years) to the remaining ‘working age’ population is generally quantified through the ‘age dependency ratio’^5,9,10^. A declining share of children and/or elders and an increase in the working-age adult population lowers the dependency ratio and can lead to opportunities for development^11,12^. For example, households with a lower number of dependents tend to increase the share of savings and to invest in the human capital of their children^13,14^, and countries experiencing a rise in the number of working-age people can expand their productivity^15^. In contrast, experiencing high young-age and old-age dependency ratios means that working age populations face a greater burden in supporting and providing the social services needed by children and elders^16^. In areas such as the sub-Saharan Africa and South Asia, age dependency ratios remain relatively high, and only in some cases have they started to slowly decline, prompting opportunities for development and economic growth^17,18^, with the potential to enter into the so-called ‘demographic dividend’^19^.

Age-sex pyramids and dependency ratios are generally measured, mapped and summarised at national levels, in spite of the fact that large subnational demographic heterogeneities exist within countries. Studies have identified and explored the role of subnational or household-level dependency ratios in some countries^20,21,22^, but these have focussed mostly on high income regions^23^. However, subnational demographic data are becoming increasingly available for countries in low income regions^24^ and this trend is being encouraged for highlighting inequalities, targeting populations at risk^24^, and guiding the development of targeted policies for the Sustainable Development Goals^25^.

Contemporary and spatially detailed datasets that identify areas where policy relevant age groups (e.g., children under the age of five, young persons, women of childbearing age and the elderly) reside are a fundamental prerequisite for monitoring demographic changes^15^, supporting geographic targeting of policies (www.undatarevolution.org), planning vaccination interventions^26^), identifying age groups at risk of mortality and morbidity^27^ and allocating resources effectively^28,29^. Accurately depicting the distribution of young age and old age dependency ratios at subnational levels aids in identifying opportunities for economic growth^18^ and possible gains from the demographic dividend^30^. It also helps in investigating other socio-economic conditions usually related to high dependency ratios, such as high fertility rates^31^, low levels of child enrolment in schools^32^ and low opportunities for productive employment^33^.

**Figure 1:**
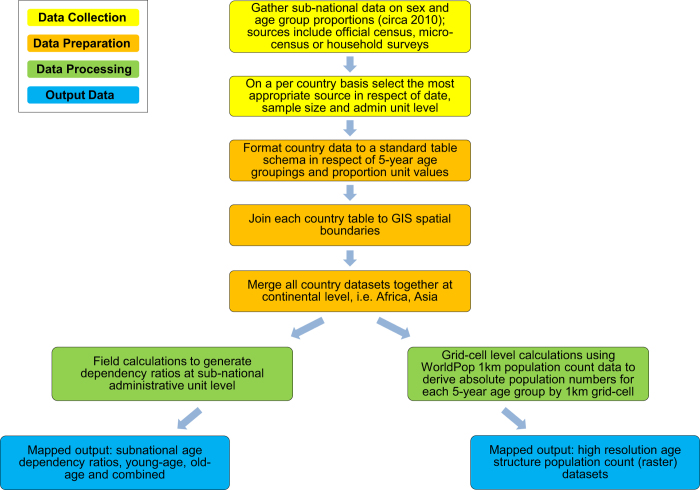
Schematic overview of the data processing method adopted to generate the WorldPop gridded subnational dependency ratio datasets and high resolution gridded 5-year age/sex group count datasets.

In the framework of the WorldPop Project (http://www.worldpop.org)^34^, advancing the approach described in Tatem *et al.*^24^, an open access archive of (i) gridded subnational dependency ratio datasets and (ii) high resolution gridded 5-year age/sex group count datasets, for 87 countries in Africa and Asia (excluding Middle Eastern countries and Japan), has been created using subnational data on population age and sex structures for circa 2010 and the corresponding WorldPop gridded continental population count datasets (Fig. 1). In particular, over 100 million records were extracted from census data, census microdata and household survey data, assembled and used to derive the proportional age and sex structures for 6,389 subnational administrative units.

## Methods

Subnational tabular data on population age and sex structures were gathered on a country by country basis. The table format and structure varied significantly from one country to the next, reflecting the wide range of data sources utilised in this project. For example, some countries presented absolute counts per age group whilst others presented proportions. The thresholds that determined the age groups also varied from country to country, ranging from 1 to 5 years. In addition, some datasets represented the full population (e.g., national census data), whilst others were a sub-sample of the population (e.g., micro-census, household surveys). These challenges required a process of standardisation aimed at harmonizing these disparate data sources into a set of consistent tables in which each row represents a subnational administrative unit and the fields contain the corresponding proportion of males, females and people in each 5-year age group (from aged 0 to 4 up to a final group aged 65 and over). Standardized country data tables were subsequently joined to their respective subnational spatial boundaries and the spatial datasets generated were then merged together to produce two continental vector datasets (i.e., Africa and Asia) depicting the proportional age and sex structure in each subnational administrative unit.

Subnational proportional age structures were then used to calculate combined, young-age and old-age dependency ratios for each administrative unit in Africa and Asia. According to the standard definitions most widely found in the literature^10,35^, the combined dependency ratio was measured as the ratio of dependents younger than 15 and aged 65 and over to the population aged 15–64; the young-age dependency ratio as the ratio of dependents younger than 15 to the working-age population aged 15–64; and the old-age dependency ratio as the ratio of dependents aged 65 and over to the working-age population. Eventually, all vector subnational dependency ratio datasets were rasterized at a resolution of 30 arc seconds (approximately 1 km at the equator).

A series of gridded 5-year age/sex group count datasets for Africa and Asia, with a spatial resolution of 30 arc seconds, were generated by gridding the subnational proportional age and sex structure datasets and overlying them with the corresponding WorldPop gridded continental population count dataset (http://www.worldpop.org.uk/data/data_sources/); with the latter adjusted to match United NationsPopulation Division (UNPD) estimates for 2010^36^.

### Data collection

Data on population age and sex structure, as close as possible to 2010, were collected at the finest spatial level available for all African and Asian countries listed in Tables 1 and 2. Where data for a given country were available from multiple sources, in addition to the year in which the data were collected, another key determinant, such as the sample size of the data, was considered in order to decide which data should be used.

The most reliable, complete and accurate source for subnational population composition is usually represented by country-level census-based data that cover the whole country population. Thus, for those countries where age and sex structure data were available from a recent national census, these were collected at the highest administrative level available, along with their corresponding spatial boundaries.

For those countries where recent full census data were not available, alternative sources were sought. Priority was given to census microdata obtained from the Integrated Public Use Microdata Series International (IPUMSI) database^37^. Census microdata represent household-level records derived from census data by sampling a representative fraction of the population (with sample size generally between 2 and 15% of the full census).

Where neither full census nor census microdata close to 2010 were available, national household survey data were obtained from the Demographic and Health Surveys (DHS), DHS special Malaria Indicators Surveys (MIS) and Aids Indicator Surveys (AIS)^38^, Social Indicator Surveys (SIS)^39^ or Multiple Indicator Cluster Surveys (MICS)^40^. Whilst providing a representation of the population closer to 2010, compared to outdated full census and census microdata, household surveys tend to suffer from a constrained sampling framework, with sample sizes being typically less than 1% of the national population and full range extending from 0.04 to 12%. Nevertheless, household survey data are designed to be representative at both national and subnational levels (typically administrative level 1, generally equivalent to provinces) and thus can be used to derive the corresponding age and sex structures. Household surveys were prioritised by survey year and sample size.

Tables 1 and 2 list data sources on a country-by-country basis for Africa and Asia, respectively, and provide information on the year of data collection and the subnational administrative level at which they were collected. In summary, the data for 30 of the 87 countries were derived from full national population and housing censuses, 17 from IPUMSI census microdata, 19 from traditional DHS, 4 from DHS-MIS/AIS/SIS and 8 from MICS (Tables 1 and 2).

For the remaining 9 countries where subnational data on population age and sex structures were not available (namely Libya, Eritrea, Western Sahara, Equatorial Guinea, Brunei, Myanmar, Papua New Guinea, Sri Lanka and Turkmenistan) 2010 UNPD country level estimates^35^ were used.

### Data preparation

Depending on the format and structure of the raw tables containing the age and sex data for each given country, a specific processing technique was developed and applied in order to standardise all data tables across all countries. Table 3 presents an example of a standardised table, for Bhutan, in which each record represents a subnational administrative unit and the fields contain the corresponding proportionate values of people (both sexes) in each 5-year age group, males and females. There follows a summary of the main techniques employed to process raw table data, summarised by data source.

#### Processing national census data

National census data is recorded and documented according to protocols determined by national governments; hence a wide range of different table data formats and structures needed to be processed and standardized. Microsoft Excel was used to manually restructure the raw data tables into a common format, structure and schema, similar to the one presented in Table 3.

#### Processing IPUMSI data

Unlike data derived from full censuses, IPUMSI data tables are already provided in a standard format and structure and thus a model was developed and applied in order to automate the processing of standardizing them. Raw country-level data are provided as comma-separated values (CSV) tables with accompanying spatial boundaries. Each CSV table is structured according to one row per person surveyed and the person’s age and sex is recorded in two fields in the same table. The raw table data were processed as follow:

- Convert each CSV table to a file geodatabase table
- Create new fields for each 5-year age group and sex class and populate with binary values, 0 or 1; a value of 1 in an age group field would indicate that the person's age fell in that range
- Use a summarise function to sum the new fields by administrative unit (note, a ‘count’ of all records by administrative unit also provides the total sampled population)
- Create and populate new fields for proportion of total population within each 5-year age group and sex class, by administrative unit
- Perform QA/QC to ensure that for each administrative unit a sum of people in all 5-year age groups equates to total population
- Perform QA/QC to ensure that for each administrative unit a sum of male and female equates to total population
- Join new table data to spatial boundaries based on administrative unit unique IDs
- Export the temporary join to a geodatabase feature class

The ArcGIS Toolbox containing the ArcGIS ModelBuilder models was used to perform all tasks described above and is distributed as part of the WorldPop-DepRatioAgeStruc-v1 code^41^ described in the Code availability subsection below.

#### Processing household survey data

Household survey data are designed to be representative at both national and sub-national level, where the sub-national level usually corresponds to region or province, depending of the survey sample design and representativeness.

Information about age structure by region were derived from household survey data by using raw data files. Given that the format and structure of all household survey data are very similar, a data management process was developed to produce standardized outputs from the raw tables containing age and sex data. Household members’ files, which include information related to each household member's age, sex and administrative unit of residence, were accessed and downloaded from the relative web pages, namely, Measure Demographic and Health Surveys (DHS) program^38^ (which also includes MIS and AIS), UNICEF Multiple Indicator Cluster Survey (MICS)^40^ and Social Indicator Surveys (SIS)^39^. Data processing was mainly performed using the SPSS (IBM) statistical software^42^. Sampling weights were applied to the calculations in order to ensure representativeness, following the relative instructions given by the data providers’ documentation. The variable containing sampling weights was identified and made available in the appropriate SPSS format. Also, in order to account for the sampling strategy adopted by the survey, relevant variables for strata and primary sampling unit were defined in SPSS (using CSPLAN ANALYSIS). Following the DHS documentation and final report, age structures were calculated only on *de facto members*, which are generally defined as those household members who slept in the household the night before the interview. Moreover, variables in the datasets corresponding to members’ age, sex and administrative unit of residence were identified. After selecting only de facto members and weighting the data appropriately, cross tabulations were applied to calculate counts and proportions of population within each 5-year age group and sex class, by administrative unit. Finally, outputs of cross tabulations were exported into excel and reformatted in order to match the standard table schema described in the Data preparation subsection.

A template SPSS syntax file, showing the process for creating proportional age and sex structures from household survey data, is distributed as part of the WorldPop-DepRatioAgeStruc-v1 code^41^ described in the Code availability subsection below.

#### Joining standardised data tables to spatial boundaries

Once formatted to a standard table schema, subnational age and sex structure data were then joined on a country-by-country basis, using a GIS system, to their corresponding spatial boundaries; with the latter representing the administrative unit level at which the data were assembled. The ArcGIS Toolbox tool ‘Join Field’ was used for this purpose (…\data management tools.tbx\joins\join field). Note the exception for the IPUMSI data for this stage as processing that data was accomplished as part of the model workflow described in the Processing IPUMSI data subsection.

Spatial boundary datasets were obtained from a range of sources, including the GADM database^43^, the DHS Spatial Repository^44^ and multiple national statistical offices. Mismatches with subnational administrative units and topological inconsistences between national boundaries were manually corrected using a GIS system.

Supplementary Fig. 1a,b (Africa and Asia, respectively) present the boundaries of the administrative units and each unit is coloured according to the proportion of the total population sampled and used to derive the 5-year age group and sex proportions. These country-level spatial datasets were then merged into two continental vector datasets (i.e., an African and Asian dataset) using the ArcGIS ‘Merge’ tool (toolboxes\system toolboxes\data management tools.tbx\General\Merge).

### Producing subnational dependency ratio datasets

Using the two continental vector datasets described above, dependency ratios were calculated using a field calculation. The ArcGIS tool ‘Field Calculator’ was used to populate three new fields (i.e., CDR, YDR and ODR), listing subnational dependency ratio values calculated at the administrative unit level:

(1)CDR=((pc014)+(pc65)/(pc1564))×100

(2)YDR=((pc014)/(pc1564))×100

(3)ODR=((pc65)/(pc1564))×100

where CDR, YDR and ODR represent the combined, young and old dependency ratio, respectively, (pc0_14) represents the proportion of the population aged 0 to 14, (pc65) represents the proportion of the population aged over 65, and (pc15_64) represents the proportion of population aged 15 to 64.

**Figure 2:**
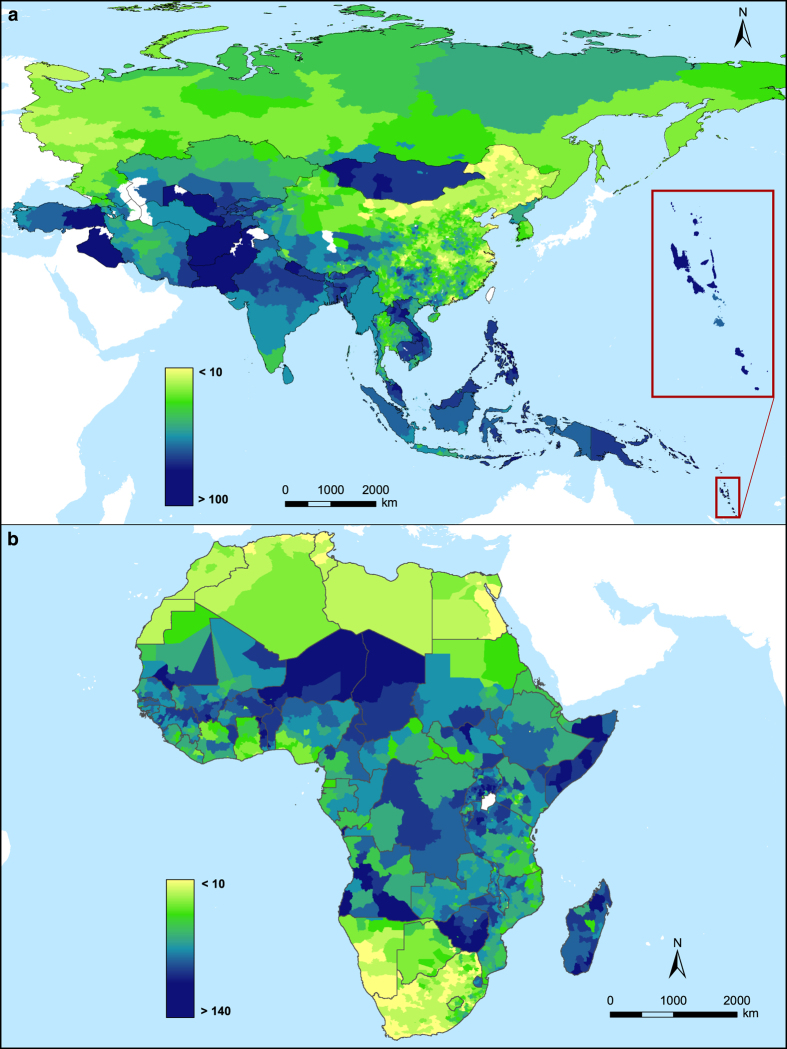
Subnational young age dependency ratio (YDR) datasets, circa 2010. (**a**) Shows dataset for Asia and (**b**) shows dataset for Africa. Note differing colour scales used between the two maps to highlight variations.

All three subnational dependency ratio vector datasets were rasterized at a resolution of 30 arc seconds (approximately 1 km at the equator). Figure 2 illustrates the spatial distribution of the subnational YDR dependency ratios in Africa and Asia, respectively.

### Producing subnational 5-year age/sex group count datasets

At this stage, to produce high resolution gridded 5-year age/sex group count datasets for 2010, the WorldPop continental gridded population count datasets for Africa and Asia (http://www.worldpop.org.uk/data/data_sources/), with the total population for each country adjusted to match United NationsPopulation Division (UNPD) estimates for 2010 (ref. 35), were multiplied by the comprehensive subnational proportional age and sex structures assembled.

The first step in this process was to convert the subnational administrative units, represented by polygon features in the two continental vector datasets described above, from vector format to raster grid format. Each polygon feature (representing a subnational administrative unit) is attributed with proportionate values for each 5-year age group and sex class (i.e., the proportion of the total population in each administrative unit belonging to each 5-year age group and sex class). The conversion process produced a stack of raster grids (one for each 5-year age group and sex class) and within each grid each pixel retained the proportionate value for that 5-year age group or sex class relating to the subnational administrative unit in which the pixel is located. Bearing in mind that subsequently these grids have to be overlaid on the WorldPop gridded continental population count datasets for Asia and Africa, in the interests of accurate grid cell calculations, the conversion process needs to ensure that the mesh of the grid is identical in terms of grid cell size (resolution) and grid cell alignment, to that of the WorldPop datasets. The conversion process uses the ArcGIS tool *Polygon to Raster*. A python script was created which called upon this tool and applied it, through iteration and whilst maintaining cell properties as described (using Geoprocessing Environment Settings), to all fields within the age/sex structure vector dataset containing the 5-year age group and male/female proportions. The ArcGIS Toolbox containing the Geoprocessing tool which calls the Python script is distributed as part of the WorldPop-DepRatioAgeStruc-v1 code^41^ described in the Code availability subsection below.

ArcGIS Model Builder was then used to automate the processing of the resulting stack of raster grids, (representing gridded subnational 5-year age group proportions). The model iterated through the stack such that each raster was sent to *Raster Calculator* tool along with the corresponding WorldPop continental gridded population count datasets (Africa or Asia) and both were utilised in a simple map algebra calculation:

(4)AGC=AGP×WPPC

(where AGC represents the resulting gridded population count dataset for a given 5-year age group, AGP is the gridded proportion dataset for the corresponding age group and WPPC is the WorldPop continental gridded population count dataset either for Africa or Asia).

This model produced a series of grids for Africa and Asia, each one providing estimates of population count for a specific age group (circa 2010) at the grid cell level. To disaggregate them by sex, two similar models, one for males and one for females, iterated through this stack to produce sex delineated population counts for each age group (circa 2010) at the grid cell level. These two models call upon the grids for subnational male and female proportions generated at an earlier stage as previously described.

**Figure 3:**
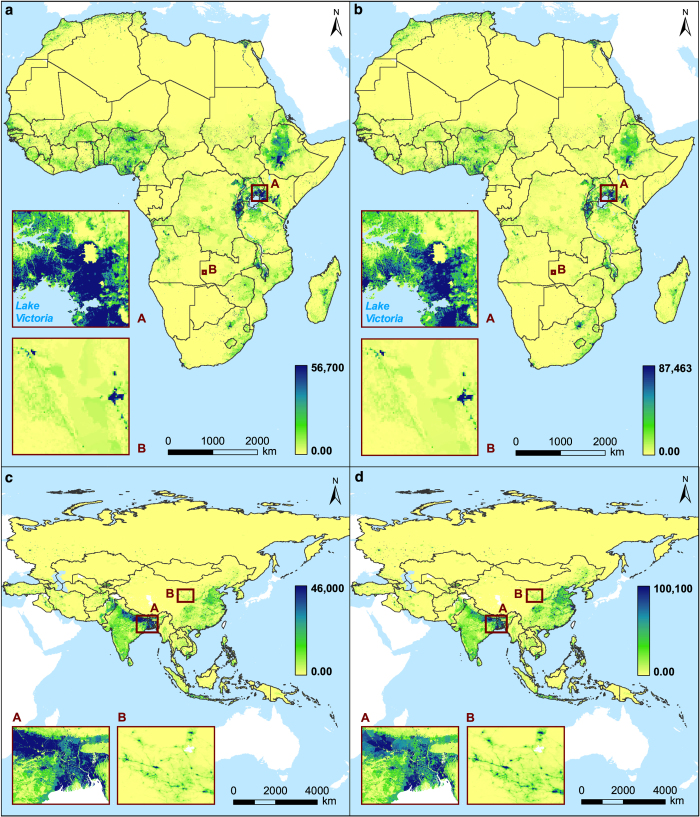
Circa 2010 High-resolution gridded population distribution presenting age structures for mainland Africa and Madagascar and the Asian region. Estimates of young age population (0 to 14yrs) are in (**a**,**c**); estimates of working age population (15 to 65) are in (**b**,**d**). The grid cell resolution is 30 arc seconds (approximately 1 km at the equator) and coordinate reference system is GCS WGS 1984.

Figure 3 represents an application of these datasets, providing gridded estimates of both young and working age population distribution (aged 0 to 14 and 15 to 65 age, respectively) for Africa and Asia.

The ArcGIS Toolbox containing the ArcGIS ModelBuilder models used to perform the steps described above is distributed as part of the WorldPop-DepRatioAgeStruc-v1 code^41^ described in the Code availability subsection below.

### Code availability

The WorldPop-DepRatioAgeStruc-v1 code^41^, used to produce the datasets described in this article, is publicly available through Figshare. It consists of (1) an ArcToolbox Geoprocessing Tool to pre-process the raw IPUMSI data, (2) an SPSS (IBM version 22) script to pre-process the DHS raw data and (3) an ArcToolbox Geoprocessing Tool to generate gridded age group structure and sex class proportion datasets and combine them with gridded population count datasets to produce the gridded age/sex structure count datasets. All of them are internally documented in order to both briefly explain their purpose and, when required, guide the user through their customization.

## Data Records

All datasets described in this article, for the 87 countries listed in Tables 1 and 2, are publicly and freely available through both the WorldPop Dataverse Repository (Data Citation 1, Data Citation 2, Data Citation 3, Data Citation 4) and the WorldPop website (http://www.worldpop.org.uk/data/data_sources/). While the datasets available through the WorldPop Dataverse Repository will be preserved in their published form while the ones available through the WorldPop website will be integrated with additional countries (Middle Eastern countries and Japan). Furthermore, additional gridded subnational dependency ratio datasets and high resolution gridded 5-year age/sex group count datasets for all countries located in Latin America and the Caribbean will be soon available through the WorldPop website.

Gridded subnational dependency ratio datasets and high resolution gridded 5-year age/sex group count datasets for each country listed in Tables 1 and 2 can be obtained by (i) downloading the corresponding datasets associated with the continent in which the country of interest is located and (ii) using the accompanying gridded ISO country code mask^45^ to extract them (Table 4).

## Technical Validation

All data collected, assembled and used were (i) already validated by the corresponding data collector, owner and/or distributor, and (ii) further checked, in the framework of this project, to ensure that they represent true trait variation by inspecting the proportions in each age class and sex group (making sure they were within reasonable ranges on a country-by-country basis). Then, both the gridded subnational dependency ratio datasets and high resolution gridded 5-year age/sex group count datasets were produced by solely processing the input data and thus the outputs were simply verified by inspecting them for abnormal values and nonsensical results. For the high resolution gridded 5-year age/sex group count datasets, both for Africa and Asia, this was done by summing all of them into a single dataset (depicting the total numbers of people for all age groups and both sexes at the grid cell level) and then subtracting it from the corresponding WorldPop continental gridded population count dataset to make sure that country totals matched the UNPD estimates for 2010. Subnational dependency ratios were also evaluated by examining their spatial distribution and by comparing them against existing UN country-level estimates.

### Comparing sub-national dependency ratios against national estimates

**Figure 4:**
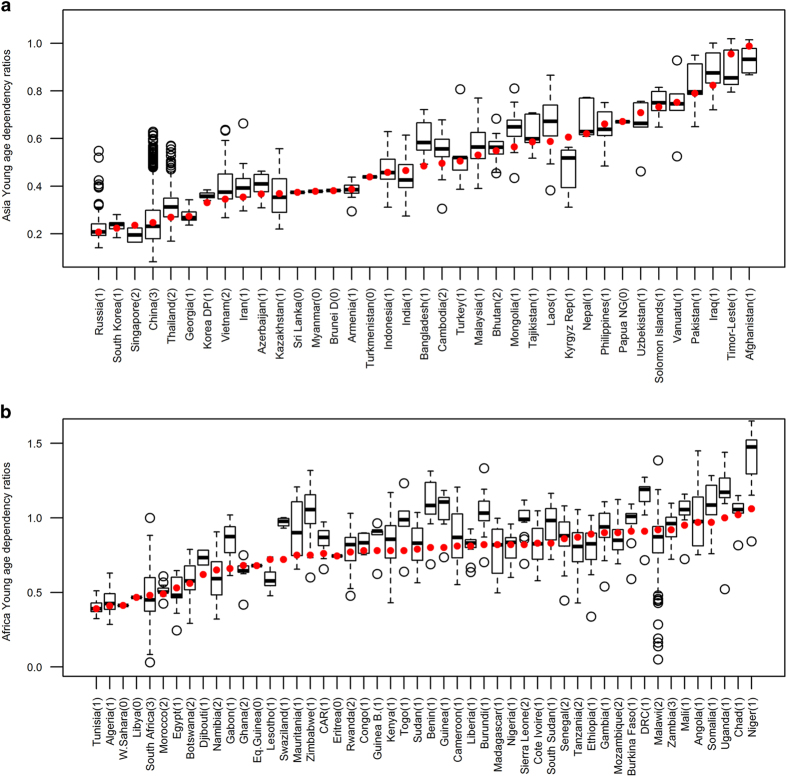
Differences between national and subnational young-age dependency ratios (YDR) for Asian and African countries. (**a**) shows Asian countries and (**b**) shows African countries. For each country, the year of World Bank national estimates (red dots) correspond to the year of our data. The solid bold center line of the boxplot shows the median values, the box width represents the interquartile range, and the whiskers extend to 1.5 times the interquartile range from the box (values further away than this are shown as open circles). The administrative unit level of the sub-national data used here is shown in brackets next to the country name on the x-axis.

The distributions of the YDR at sub-national scales for Africa and Asia were plotted against World Bank national estimates, for each country’s correspondent year. Figure 4 gives an indication of the size of the sub-national variation in the YDR shown in the subnational level datasets that is masked when averaging at national levels.

The variation observed is related also to the administrative unit level of the input data, with those countries for which the most spatially detailed YDR were available (i.e., Tanzania, South Africa and China) showing the largest differences between minimum and maximum estimates. Nevertheless, even for those countries where subnational data were only available at administrative unit level 1, sub-national differences from the World Bank national level estimates are evident. Additionally, national estimates are often highly influenced by areas with high density of population whose rates greatly influence the averages, and this is reflected by the World Bank estimates falling outside the interquartile range of the boxplots in many cases (Fig. 4).

### Sensitivity tests on differences over time in population structures

All available sources that were as close as possible to 2010 at the time of data processing were considered in this work. However, for some countries only relatively outdated data were available. To test the assumption of no major changes in age structures between years, the absolute change (difference) in age group specific percentage points was tested, in countries where two or more comparable time points were available from household surveys,. Two countries in Asia and one country in Sub Saharan Africa were selected, namely: Nepal (DHS Surveys 2011–2006 & 2011–2001), Kazakhstan (MICS Surveys 2011–2006) and Guinea (DHS Surveys 2011–2005 & 2011–1999). Absolute change (difference) in percentage points was calculated for each survey region and total country (Table 5) (available online only).

Also, for the same countries and all different time points, population pyramids were constructed and structures between years were compared (Supplementary Figs 2a–4c). Population pyramids constructed for this test follow the methodology described in the Methods section, therefore they reflect the input data utilised to construct the datasets described in this article. In order to be consistent with the method applied here, country level proportions of males and females were applied for all age groups. Results from our tests suggest that overall, differences are relatively small. Through looking at country totals and comparing pyramids, the highest differences in percentage points are shown for younger age groups, and this pattern is consistent among all tested countries. Furthermore, country level tests for Kazakhstan also show that some older age groups (60–64 and 65–69) present larger differences in percentage points, compared to the other age groups. At subnational level, the most consistent differences are shown in Central, Far-western regions in Nepal when comparing the 2001 to the 2011 survey, and in Mangystau oblysy (Kazakhstan 2011–2006) for age group 0–5, when compared to other regions and age classes.

## Usage Notes

The gridded subnational dependency ratio datasets and high resolution gridded 5-year age/sex structure datasets described in this article can be used to support a range of applications, from planning interventions to designing strategies and deriving health/development metrics, and to predict response variables that are intrinsically dependent on dependency ratios and age/sex structures.

Ongoing work involves the integration of these datasets with information related to health and development for planning interventions. This can include, for example, integration with information related to vaccination rates of children under the age of five^26^, access to antenatal, delivery and postnatal care for women of reproductive age^46^, and disease prevalence and burden^47,48^.

These datasets can also be used as a base for measuring demographic progress in relation to a set of topics like, ageing, population growth, dynamics and projections^5,6,8,15,49,50^. Furthermore, they can be combined with economic data to assess the association between the population pyramid and the economic development in a given country, as well as to study the effects of population structure on savings and growth^51^. Finally, age and sex compositions can be used to determine the demographic events that will occur and their impact on the types of facilities and services (e.g., schools and maternity services) needed by the population.

Although the subnational dependency ratio and age/sex structure datasets presented here represent the best available datasets at this level of spatial detail/resolution, it is important to highlight that limitations still exist. Indeed, all of the census data, census microdata and survey-based data used here are subject to various sources of error and bias.

Surveys usually do not sample from certain groups and places (e.g., indigenous groups, informal settlements, places experiencing civil unrest and refugee camps) either because of political biases, missing sampling frames, or security issues. Therefore, these groups are not covered in surveys, and in some cases are also not included in national censuses. Household survey data are in most cases sampled using complex survey design procedures^52^ and estimates derived from household survey datasets are usually representative at administrative level 1 or provinces, or a combination of those. IPUMS-International microdata are samples from population censuses from National Statistical Offices taken around the world since 1960. Sample data are derived by using different sampling designs (https://international.ipums.org/international/variance_estimation.shtml). Furthermore, they cover only a fraction of the population (approximately 5%) and, as for every sample, they are subject to sampling errors^37^.

Uncertainties also arise over comparisons being made between primarily full census-based estimates of dependency ratios and age/sex structures and those based on IPUMSI microdata and household survey data. Differences between the way these were measured contributes to uncertainties in comparisons, though strong correlations between household survey-derived age structures, and those derived from census data suggest that such differences may be small^24^. For some countries, the input data used in this project were also relatively outdated and/or coarse—whilst the precision with which heterogeneities in dependency ratios and age/sex structures are mapped is improved over simple country-level estimates, the datasets presented here are still limited through e.g., only one set of values for Libya and thousands for China. Moreover, like most other population characteristics reported at the administrate unit level, the dependency ratios and age/sex structures are also subject to the modifiable areal unit problem^53^.

Population data that were sex disaggregated by age group at subnational administrative unit level were only available for a minority of countries. In most cases, the subnational level data were disaggregated only by age group with a single two-way sex split. Therefore in order to maintain the comparability across countries, the subnational level sex split was simply applied across all age groups within the corresponding subnational administrative unit.

## Additional Information

**How to cite this article:** Pezzulo, C. *et al.* Sub-national mapping of population pyramids and dependency ratios in Africa and Asia. *Sci. Data* 4:170089 doi: 10.1038/sdata.2017.89 (2017).

**Publisher’s note:** Springer Nature remains neutral with regard to jurisdictional claims in published maps and institutional affiliations.

## Supplementary Material



Supplementary Information

## Figures and Tables

**Table 1:** Data sources for African countries from which age and sex proportions were derived.

**Country**	**Data type used**	**Year**	**Administrative unit level**	**Data source**
Algeria	Census	2008	1	Office National des Statistique, Algeria
Angola	MIS	2011	1	MEASURE Demographic and Health Surveys, USAID
Benin	DHS	2011	1	MEASURE Demographic and Health Surveys, USAID
Botswana	Census	2006	2	Central Statistics Office, Botswana
Burkina Faso	Census	2006	1	Institut National de la Statistique et de la Demographie (INSD), Burkina Faso
Burundi	DHS	2010	1	MEASURE Demographic and Health Surveys, USAID
Cameroon	DHS	2011	1	MEASURE Demographic and Health Surveys, USAID
Central African Republic	MICS	2006	1	UNICEF
Chad	DHS	2004	1	MEASURE Demographic and Health Surveys, USAID
Congo	AIS	2009	1	MEASURE Demographic and Health Surveys, USAID
Congo, Democratic Republic	DHS	2013	1	MEASURE Demographic and Health Surveys, USAID
Cote d'Ivoire	AIS	2005	1	MEASURE Demographic and Health Surveys, USAID
Djibouti	MICS	2006	1	UNICEF
Egypt	Census microdata	2006	1	Integrated Public Use Microdata Series, International (IPUMSI)
Equatorial Guinea	UN	2010	0	World Population Prospects, United Nations Population Division
Eritrea	UN	2010	0	World Population Prospects, United Nations Population Division
Ethiopia	Census	2007	1	Central Statistical Agency of Ethiopia
Gabon	DHS	2012	1	MEASURE Demographic and Health Surveys, USAID
Gambia	MICS	2006	1	MEASURE Demographic and Health Surveys, USAID
Ghana	Census	2010	2	United States Census Bureau, USAID
Guinea	DHS	2012	1	MEASURE Demographic and Health Surveys, USAID
Guinea-Bissau	MICS	2006	1	UNICEF
Kenya	Census	2010	1	United States Census Bureau, USAID
Lesotho	Census	2004	1	Lesotho Bureau of Statistics
Liberia	Census	2008	1	Liberia Institute of Statistics and Geo-Information Service
Libya	UN	2010	0	World Population Prospects, United Nations Population Division
Madagascar	DHS	2009	1	MEASURE Demographic and Health Surveys, USAID
Malawi	Census	2008	2	National Statistical Office, Malawi
Mali	DHS	2012	1	MEASURE Demographic and Health Surveys, USAID
Mauritania	MICS	2007	1	UNICEF
Morocco	Census	2004	2	Haut Commissariat au Plan, Morocco
Mozambique	Census	2007	2	Instituto Nacional de Estatistica, Mozambique
Namibia	Census	2010	2	United States Census Bureau, USAID
Niger	DHS	2012	1	MEASURE Demographic and Health Surveys, USAID
Nigeria	Census	2006	1	National Bureau of Statistics, Nigeria
Rwanda	Census	2010	2	United States Census Bureau, USAID
Senegal	Census microdata	2002	2	Integrated Public Use Microdata Series, International (IPUMSI)
Sierra Leone	Census	2004	2	Statistics Sierra Leone
Somalia	MICS	2006	1	UNICEF
South Africa	Census	2010	3	United States Census Bureau, USAID
South Sudan	Census microdata	2008	1	Integrated Public Use Microdata Series, International (IPUMSI)
Sudan	Census microdata	2008	1	Integrated Public Use Microdata Series, International (IPUMSI)
Swaziland	DHS	2007	1	MEASURE Demographic and Health Surveys, USAID
Togo	DHS	2013	1	MEASURE Demographic and Health Surveys, USAID
Tunisia	Census	2004	1	National Statistics Institute, Tunisia
Uganda	DHS	2011	1	MEASURE Demographic and Health Surveys, USAID
United Rep. of Tanzania	Census	2010	2	United States Census Bureau, USAID
Western Sahara	UN	2010	0	World Population Prospects, United Nations Population Division
Zambia	Census	2010	3	United States Census Bureau, USAID
Zimbabwe	DHS	2011	1	MEASURE Demographic and Health Surveys, USAID
Notes: AIS, Aids Indicator Survey; DHS, Demographic and Health Survey; MICS, Multiple Indicator Cluster Survey; MIS, Malaria Indicators Survey; the administrative unit level number in the 3rd column refers to the types of administrative divisions used for each country (with size of the administrative units decreasing from level 0, corresponding to the whole country, to level 3; https://en.wikipedia.org/wiki/List_of_administrative_divisions_by_country).				

**Table 2:** Data sources for Asian countries from which age and sex proportions were derived.

**Country**	**Data type used**	**Year**	**Administrative Unit Level**	**Data source**
Afghanistan	DHS	2010	1	MEASURE Demographic and Health Surveys, USAID
Armenia	Census microdata	2001	1	Integrated Public Use Microdata Series, International (IPUMSI)
Azerbaijan	DHS	2006	1	MEASURE Demographic and Health Surveys, USAID
Bangladesh	DHS	2011	1	MEASURE Demographic and Health Surveys, USAID
Bhutan	Census	2005	2	National Statistics Bureau, Bhutan
Brunei Darussalam	UN	2010	0	World Population Prospects, United Nations Population Division
Cambodia	Census	2010	2	United States Census Bureau, USAID
China	Census	2010	3	National Bureau of Statistics of the People's Republic of China
Georgia	MICS	2005	1	MEASURE Demographic and Health Surveys, USAID
India	Census	2010	1	United States Census Bureau, USAID
Indonesia	Census	2010	1	United States Census Bureau, USAID
Iran	Census microdata	2006	1	Integrated Public Use Microdata Series, International (IPUMSI)
Iraq	Census microdata	1997	1	Integrated Public Use Microdata Series, International (IPUMSI)
Kazakhstan	MICS	2011	1	MEASURE Demographic and Health Surveys, USAID
Korea, Democratic People's Republic of	Census	2010	1	National Bureau of Statistics, North Korea
Kyrgyz Republic	Census microdata	1999	1	Integrated Public Use Microdata Series, International (IPUMSI)
Laos	SIS	2012	1	MEASURE Demographic and Health Surveys, USAID
Malaysia	Census microdata	2000	1	Integrated Public Use Microdata Series, International (IPUMSI)
Mongolia	Census microdata	2000	1	Integrated Public Use Microdata Series, International (IPUMSI)
Myanmar	UN	2010	0	World Population Prospects, United Nations Population Division
Nepal	DHS	2011	1	MEASURE Demographic and Health Surveys, USAID
Pakistan	Census microdata	1998	1	Integrated Public Use Microdata Series, International (IPUMSI)
Papua New Guinea	UN	2010	0	World Population Prospects, United Nations Population Division
Philippines	Census microdata	2000	1	Integrated Public Use Microdata Series, International (IPUMSI)
Russia	Census	2010	1	United States Census Bureau, USAID
Singapore	Census	2010	2	Statistics Singapore, Singapore
Solomon Islands	Census	2009	1	Solomon Islands National Statistics Office, Solomon Islands
South Korea	Census	2010	1	Statistics Korea, South Korea
Sri Lanka	UN	2010	0	World Population Prospects, United Nations Population Division
Tajikistan	DHS	2012	1	MEASURE Demographic and Health Surveys, USAID
Thailand	Census	2010	2	United States Census Bureau, USAID
Timor-Leste	DHS	2010	1	MEASURE Demographic and Health Surveys, USAID
Turkey	DHS	1998	1	MEASURE Demographic and Health Surveys, USAID
Turkmenistan	UN	2010	0	World Population Prospects, United Nations Population Division
Uzbekistan	DHS	1996	1	MEASURE Demographic and Health Surveys, USAID
Vanuatu	Census	2009	1	Vanuatu National Statistics Office (VNSO), Vanuatu
Vietnam	Census microdata	2009	2	Integrated Public Use Microdata Series, International (IPUMSI)
Notes: AIS, Aids Indicator Survey; DHS, Demographic and Health Survey; MICS, Multiple Indicator Cluster Survey; MIS, Malaria Indicators Survey; the administrative unit level number in the 3rd column refers to the types of administrative divisions used for each country (with size of the administrative units decreasing from level 0, corresponding to the whole country, to level 3; https://en.wikipedia.org/wiki/List_of_administrative_divisions_by_country).				

**Table 3:** Example of a standardised country table containing the proportionate values of people (both sexes) in each 5-year age group, males and females in each administrative unit.

**NAME**	**ADM_NAME**	**MPROP**	**FPROP**	**A0004**	**A0509**	**A1014**	**A1519**	**A2024**	**A2529**	**A3034**	**…**	**A65PL**
Bhutan	BUMTHANG	54.3	45.7	8.5	9.9	12.4	12.1	12.1	9.2	6.4	…	6.2
Bhutan	CHHUKHA	56.9	43.1	9.4	10.5	10.7	10.6	13.4	11.7	8.2	…	2.6
Bhutan	TSIRANG	51.0	49.0	10.4	10.6	12.9	11.9	9.7	7.9	5.9	…	5.4
Bhutan	DAGANA	50.3	49.7	11.1	13.1	14.4	12.4	8.0	7.1	5.9	…	4.6
Bhutan	GASA	52.5	47.5	10.4	11.5	10.8	9.1	10.5	9.4	7.8	…	5.4
Bhutan	SARPANG	52.1	47.9	10.6	11.7	12.0	11.4	11.1	9.4	6.9	…	3.7
Bhutan	HAA	53.9	46.1	8.5	10.6	12.6	12.8	10.1	9.0	6.9	…	4.9
Bhutan	LHUENTSE	50.2	49.8	10.3	12.8	12.4	12.4	7.5	7.7	5.8	…	6.8
Bhutan	MONGGAR	50.4	49.6	10.3	11.8	12.8	12.8	9.0	7.8	6.2	…	5.6
Bhutan	PARO	53.0	47.0	8.4	10.0	11.4	11.9	12.6	9.6	7.1	…	5.3
Bhutan	PEMAGATSHEL	49.5	50.5	9.8	11.3	12.6	13.6	7.4	6.5	4.6	…	8.1
Bhutan	PUNAKHA	50.7	49.3	9.7	11.6	11.8	14.8	9.1	7.5	6.0	…	5.4
Bhutan	SAMTSE	52.1	47.9	10.0	11.3	12.5	10.8	10.6	8.7	6.8	…	4.9
Bhutan	SAMDRUPJONGKHAR	51.4	48.6	10.6	11.8	12.8	11.0	9.3	8.4	6.3	…	4.9
Bhutan	ZHEMGANG	50.9	49.1	9.7	11.6	14.0	13.6	8.8	6.7	5.4	…	6.5
Bhutan	TRASHIYANGTSE	49.9	50.1	10.9	12.4	13.0	12.3	8.1	6.8	5.4	…	5.3
Bhutan	TRASHIGANG	51.0	49.0	9.9	11.9	13.1	13.1	9.4	6.9	5.7	…	5.6
Bhutan	THIMPHU	54.2	45.8	9.6	9.6	10.9	11.6	15.3	11.4	7.9	…	3
Bhutan	TRONGSA	51.2	48.8	9.9	11.8	12.5	11.6	9.2	8.1	6.1	…	6.9
Bhutan	WANGDUE	51.7	48.3	10.1	11.5	12.3	11.3	10.1	8.1	6.6	…	5.7
(Note that the table is for illustrative purposes only, and hence only displays age groups up to 34 years of age and 65 plus).												

**Table 4:** Name, description (from XX to YY represents all 5-year age groups from 00 up to a final group aged 65 and over), format, resolution and DOI of all datasets available for Africa and Asia (including the gridded ISO country code mask^45^ needed to extract country datasets from the continental datasets).

**Name**	**Description**	**Format/ Resolution**	**Dataverse DOI**
AFR_2010_SubNat_DepRatio	Subnational combined (CDR), young (YDR) and old (ODR) dependency ratio datasets for 50 African countries	GeoTIFF/ 3 arc seconds	10.7910/DVN/S5JHQN
ASIA_2010_SubNat_DepRatio	Subnational combined (CDR), young (YDR) and old (ODR) dependency ratio datasets for 37 Asian countries	GeoTIFF/ 3 arc seconds	10.7910/DVN/6TPPZ8
AFR_PPP_AGE_2010	Female/male per pixel, in the age group from XX to YY, for 50 African countries in 2010; with total population for each country adjusted to match 2010 UN national estimates^39^	GeoTIFF/ 3 arc seconds	10.7910/DVN/4MJN3G
ASIA_PPP_AGE_2010	Female/male per pixel, in the age group from XX to YY, for 37 Asian countries in 2010; with total population for each country adjusted to match 2010 UN national estimates^39^	GeoTIFF/ 3 arc seconds	10.7910/DVN/GUSJUZ
WP_DemographicData_CountryMask	Numeric ISO-3166 country codes for all 87 African and Asian countries listed in Table1 an 2	GeoTIFF/ 3 arc seconds	10.7910/DVN/S5JHQN10.7910/DVN/6TPPZ810.7910/DVN/4MJN3G10.7910/DVN/GUSJUZ

**Table 5:** Absolute change (difference) in age group specific percentage points between survey round years in Nepal (DHS Surveys 2011-2006 & 2011-2001), Kazakhstan (MICS Surveys 2011-2006), Guinea (DHS Surveys 2011-2005 & 2011-1999), for each region and total country

**Country/Region**	**Age groups**																
	**<5**	**5–9**	**10–14**	**15–19**	**20–24**	**25–29**	**30–34**	**35–39**	**40–44**	**45–49**	**50–54**	**55–59**	**60–64**	**65–69**	**70–74**	**75–79**	**80+**
Nepal DHS (2011-2006)																	
Eastern	−2.00	−1.40	0.50	−0.60	−0.20	0.40	0.60	0.30	−0.10	−0.40	0.90	0.90	0.00	0.50	0.00	0.50	0.20
Central	−2.00	−1.40	0.20	0.30	0.10	−0.10	0.60	0.70	0.20	−0.50	0.90	0.50	−0.30	0.30	0.40	0.10	0.20
Western	−2.60	−2.50	0.00	0.70	−0.40	−0.20	0.10	0.60	0.40	0.50	0.90	0.90	0.70	0.50	−0.10	0.20	0.30
Mid-western	−0.30	−1.80	−0.50	−0.20	0.70	0.70	−0.10	0.10	−0.70	−0.20	0.80	0.80	−0.20	0.40	0.10	0.30	0.00
Far-western	−1.20	−0.50	0.60	−1.30	0.10	−0.60	−1.20	0.20	0.10	−0.20	0.60	0.30	1.30	0.80	0.50	0.10	0.30
**Total**	−1.90	−1.60	0.20	−0.10	0.00	0.00	0.20	0.50	0.10	−0.20	0.90	0.70	0.20	0.50	0.20	0.20	0.20
Nepal DHS (2011-2001)																	
Eastern	−3.06	−1.93	−0.50	−0.19	1.06	0.63	−0.46	0.13	−0.15	−0.10	1.42	0.95	0.60	0.14	0.33	0.60	0.57
Central	−5.35	−3.47	1.13	1.24	0.22	−0.02	0.60	0.87	1.03	0.63	0.99	0.71	0.29	0.19	0.36	0.07	0.56
Western	−3.87	−3.85	0.16	0.57	0.14	−0.27	0.38	1.04	0.53	0.39	1.04	0.93	0.87	0.42	0.50	0.51	0.56
Mid-western	−2.90	−1.67	−0.38	0.30	0.36	0.63	−0.26	0.96	0.32	−0.04	0.36	0.78	0.34	0.34	0.51	0.44	−0.09
Far-western	−4.33	−1.49	0.28	1.12	0.60	0.64	−0.62	0.53	0.38	−0.01	0.70	0.28	0.53	0.46	0.44	0.18	0.35
**Total**	−4.09	−2.74	0.23	0.61	0.47	0.25	0.06	0.71	0.49	0.26	0.99	0.78	0.52	0.27	0.41	0.35	0.47
Kazakhstan (2011-2006)																	
Akmola oblysy	0.13	−0.69	−1.81	−1.58	0.48	0.61	0.19	−0.78	−0.76	−0.51	2.44	0.39	2.07	−1.36	1.73	−0.72	0.19
Aktobe oblysy	2.90	−0.31	−1.91	−2.11	−0.08	−0.23	1.06	−0.83	0.07	−0.10	0.67	0.92	1.12	−2.21	1.41	−0.57	0.21
Almaty oblysy	0.33	0.47	−2.13	−0.29	1.44	−0.41	−0.61	−0.93	0.11	0.67	1.04	−0.05	1.00	−1.05	0.33	−0.31	0.39
Almati city	−2.82	0.99	−2.00	−1.99	2.35	1.08	0.88	2.55	−0.69	−0.17	−1.89	−0.49	2.00	−1.48	0.83	0.36	0.49
Astana city	2.06	0.75	−2.11	−0.83	1.60	0.72	−0.04	−0.11	−1.26	−0.28	1.57	−0.14	0.68	−1.61	0.20	−0.72	−0.49
Atyrau oblysy	3.27	0.30	−2.40	−4.20	0.34	0.31	0.67	−0.66	−0.42	0.12	2.00	1.63	1.13	−1.02	−0.49	−0.55	−0.01
Shygys-Kazakhstan oblysy	2.36	1.37	−0.77	−3.18	−1.22	−0.37	1.24	0.14	−0.22	−0.61	−0.63	1.38	1.79	−1.98	0.83	−0.64	0.51
Zhambyl oblysy	1.59	1.53	−2.83	0.04	0.43	−1.05	−0.01	−1.17	−0.32	0.46	0.85	−0.39	2.03	−0.84	0.61	−0.80	−0.11
Batys-Kazakhstan oblysy	2.74	1.51	−2.75	−3.48	−0.86	0.62	0.79	−0.21	−2.29	−0.21	0.74	1.05	1.82	−1.93	1.37	0.54	0.58
Karagandy oblysy	2.91	1.60	−0.95	−3.09	−0.14	0.43	0.91	0.65	−1.69	−0.43	−0.28	0.25	0.92	−2.63	1.58	0.04	−0.06
Kostanai oblysy	0.57	0.45	−2.08	−3.03	1.32	0.35	−1.09	−0.44	−0.01	0.76	2.90	−0.83	0.83	−2.31	2.22	−0.32	0.72
Kyzylorda oblysy	2.90	1.12	−2.46	−2.73	−1.51	−0.60	1.27	0.21	−0.53	0.20	1.29	−0.53	1.01	−0.86	1.18	0.01	0.04
Mangystau oblysy	5.29	1.38	−1.82	−1.01	−0.51	−0.21	1.40	−0.62	−1.80	−0.32	−2.13	0.60	0.46	−1.06	0.59	−0.22	−0.04
Pavlodar oblysy	1.38	−0.21	−2.28	−2.98	0.43	2.07	1.38	−0.98	−1.95	0.58	1.60	0.23	2.88	−2.55	1.00	−0.33	−0.26
Soltustik-Kazakhstan oblysy	−0.08	0.97	−1.23	−3.21	−0.23	−0.46	0.24	1.71	−1.20	−0.74	0.84	1.87	2.03	−2.40	1.70	−0.44	0.62
Ontustik-Kazakhstan oblysy	1.88	1.22	−2.03	−0.89	−0.16	−0.07	0.31	−0.98	0.25	0.11	0.73	−0.09	0.83	−1.18	0.51	−0.44	0.00
**Total**	1.68	1.02	−1.75	−1.94	0.33	0.16	0.49	−0.19	−0.64	−0.11	0.52	0.16	1.32	−1.74	0.89	−0.37	0.16
Guinea (2011-2005)																	
Basse Guinée	−1.00	−0.36	0.95	0.11	0.84	1.07	−0.43	−0.80	−0.31	−1.78	0.59	0.03	0.35	0.07	0.45	−0.05	0.29
Moyenne Guinée	0.33	0.40	−1.42	−1.17	0.67	0.74	−0.14	−1.03	−0.46	−0.17	0.46	0.10	0.54	0.20	−0.03	0.37	0.64
Haute Guinée	0.59	−1.32	0.07	1.31	1.34	0.35	0.32	−1.06	−0.12	−0.48	0.22	−0.03	−0.07	−0.35	−0.32	−0.13	−0.30
Guinée Forestiere	−0.16	0.19	0.18	0.74	0.05	0.51	−0.54	−0.50	−0.01	−0.69	0.55	−0.33	0.13	0.08	−0.06	−0.09	−0.09
Conakry	0.86	−1.93	0.91	−0.39	0.61	0.75	−0.52	−0.54	−0.49	−0.48	0.36	0.49	0.18	0.11	0.05	0.14	0.04
**Total**	−0.04	−0.62	0.18	0.22	0.82	0.80	−0.22	−0.78	−0.27	−0.79	0.43	0.00	0.21	−0.02	−0.01	0.01	0.10
Guinea (2011-1999)																	
Basse Guinée	−1.71	−2.17	1.50	−0.17	0.63	0.00	−0.85	−0.33	0.24	−0.73	1.21	0.21	0.75	0.25	0.52	0.37	0.33
Moyenne Guinée	0.75	−1.64	−1.34	−0.33	−0.06	−0.07	−0.21	−0.62	−0.23	0.70	0.63	0.45	0.32	0.51	0.03	0.62	0.53
Haute Guinée	0.36	−0.31	2.24	0.14	−0.19	−0.60	0.06	−0.60	0.32	−0.14	0.43	−0.37	−0.50	−0.19	−0.18	−0.30	−0.11
Guinée Forestiere	−0.65	−0.16	1.55	0.89	0.06	−0.66	−0.57	−0.66	0.47	−0.73	0.52	−0.18	−0.06	0.04	0.22	0.00	−0.02
Conakry	0.30	−2.36	1.31	0.00	1.29	−0.78	−0.97	−0.57	0.03	−0.87	0.95	0.49	0.92	0.09	0.04	0.25	0.11
**Total**	−0.18	−1.41	0.98	0.24	0.44	−0.27	−0.44	−0.53	0.17	−0.35	0.67	0.08	0.21	0.09	0.09	0.16	0.13
Notes: percent difference is given as %-points. DHS=Demographic and Health Survey; MICS=Multiple Indicator Cluster Survey.																	
