# Supplementary material for: Sub-national mapping of population pyramids and dependency ratios in Africa and Asia
Source: Sci Data. 2017 Jul 19;4:170089. doi: 10.1038/sdata.2017.89 (PMC5516541; doi:10.1038/sdata.2017.89)
Supplement: Supplementary Information [file sdata201789-s2.pdf]

Supplementary Figures

Table of Contents

Supplementary Figure 1a………2
Supplementary Figure 1b………3
Supplementary Figure 2a………4
Supplementary Figure 2b………5
Supplementary Figure 2c………6
Supplementary Figure 3a………7
Supplementary Figure 3b………8
Supplementary Figure 4a………9
Supplementary Figure 4b……10
Supplementary Figure 4c……11

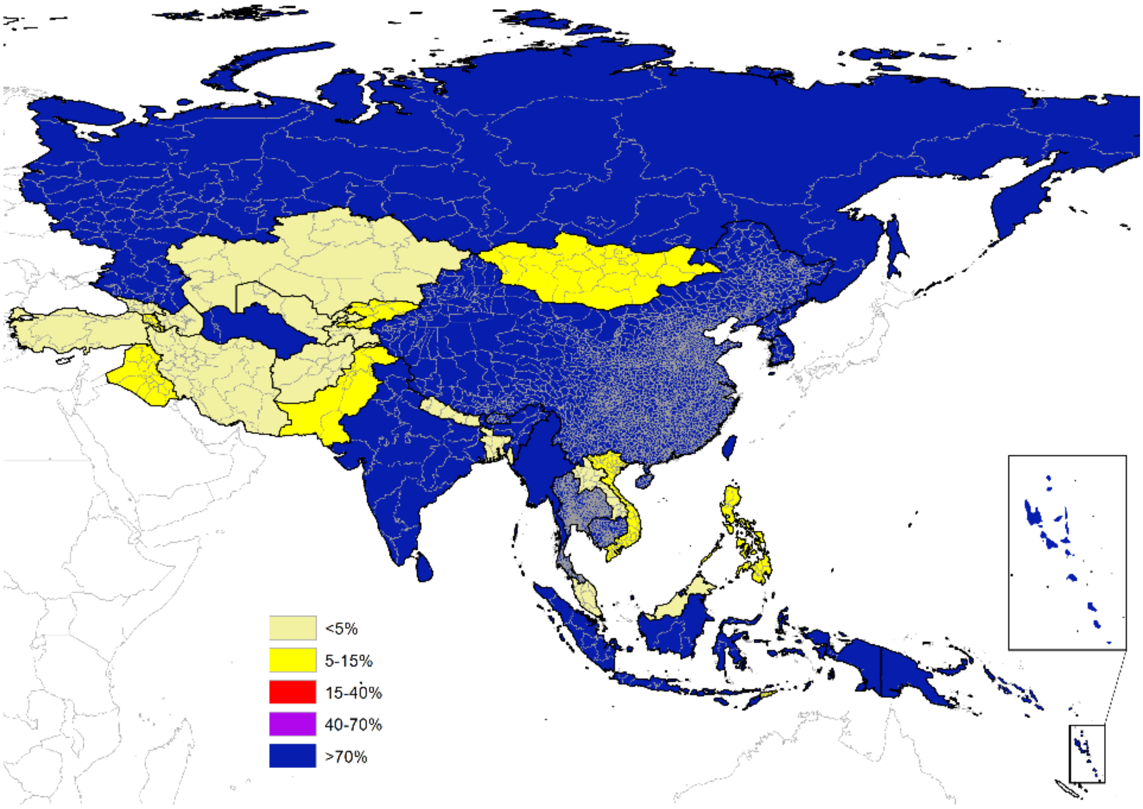

Supplementary Figure 1a. *Sample size of the sub-national age and sex proportion data used, displayed as a percentage of the estimated total sub-national unit population at the time of survey, Asia.*

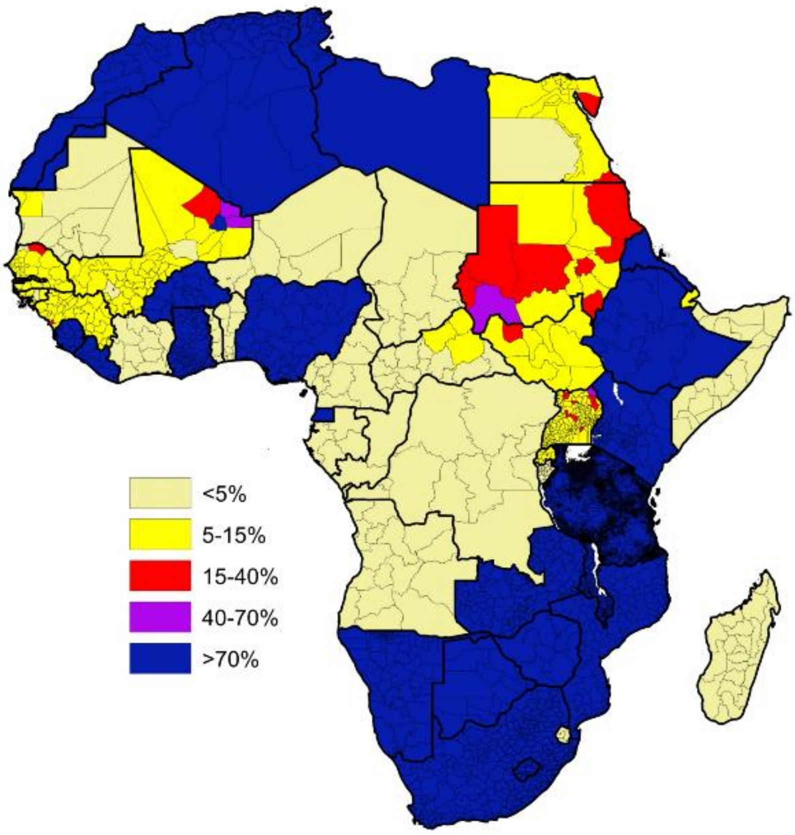

Supplementary Figure 1b. *Sample size of the sub-national age and sex proportion data used, displayed as a percentage of the estimated total sub-national unit population at the time of survey, Africa.*

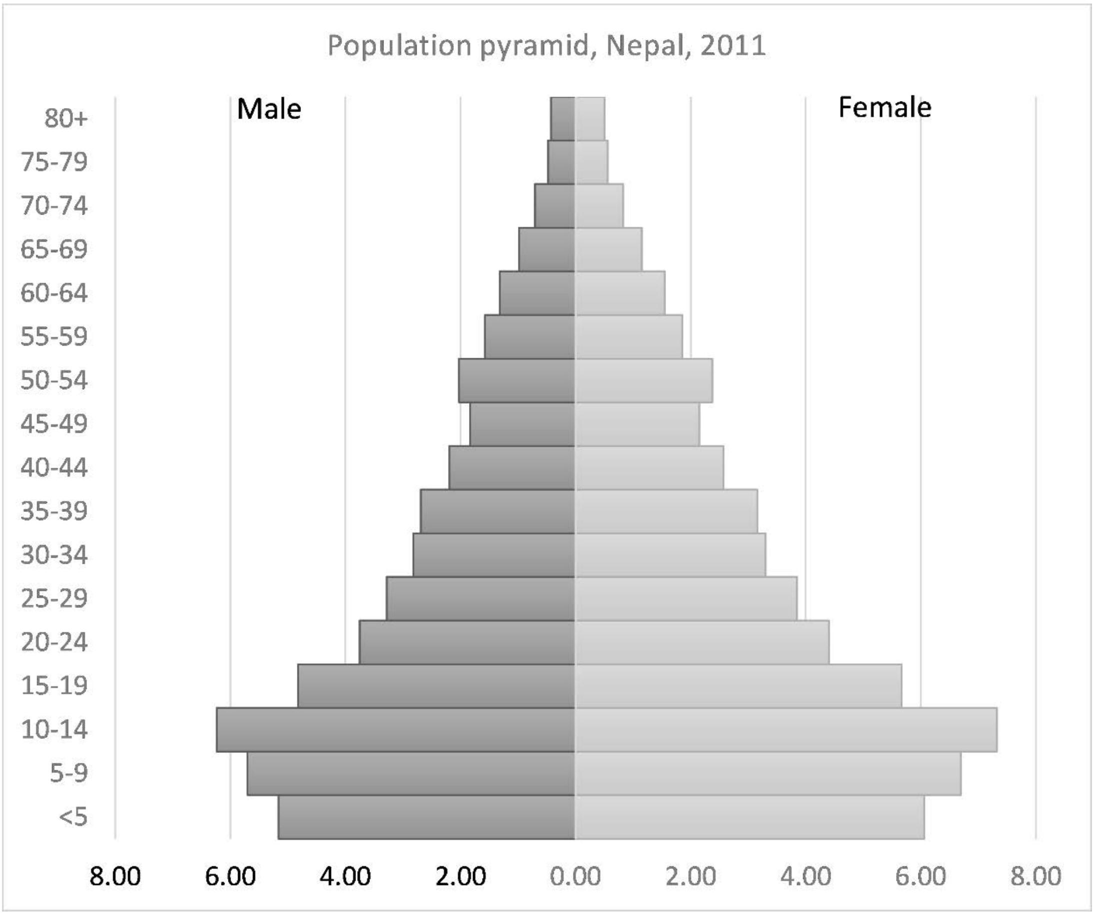

Supplementary Figure 2a. *Population pyramid, Nepal DHS, 2011. Population pyramids follow methodology as described in the manuscript (Methods section).*

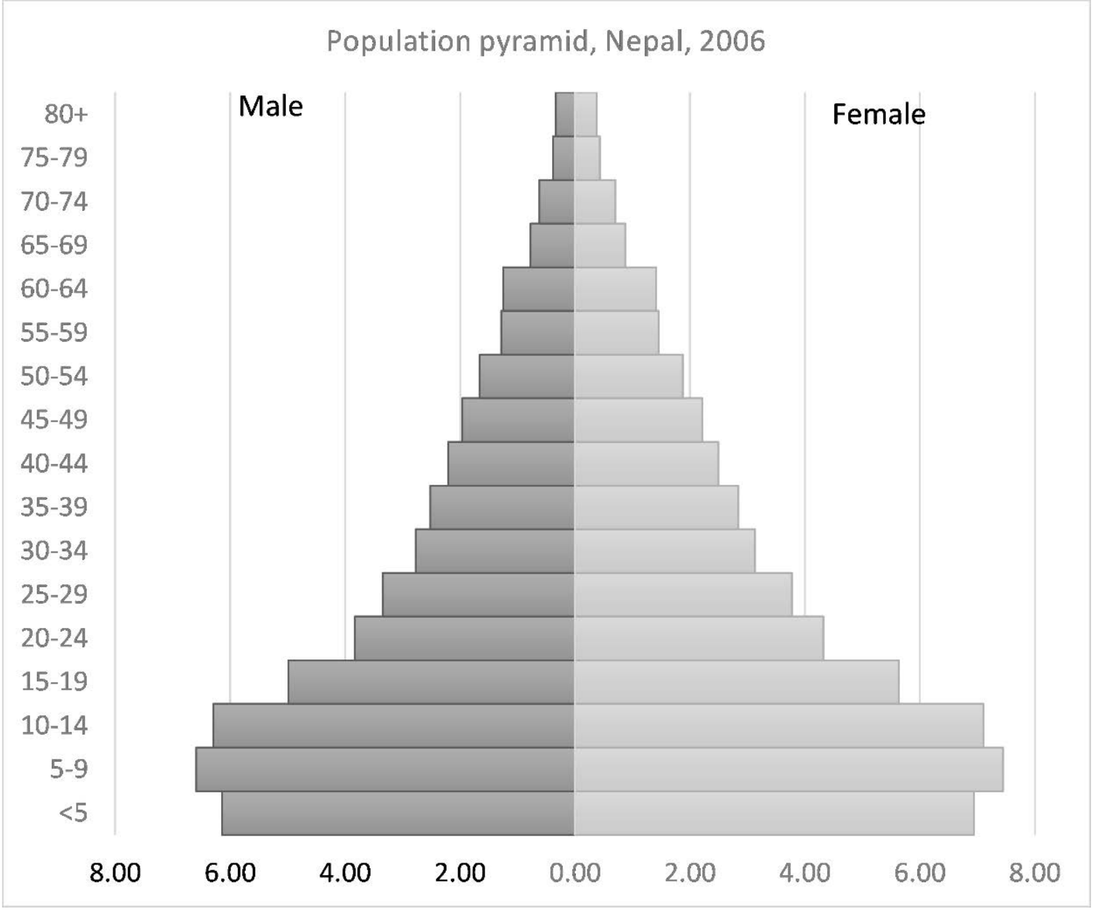

Supplementary Figure 2b. *Population pyramid, Nepal DHS, 2006. Population pyramids follow methodology as described in the manuscript (Methods section).*

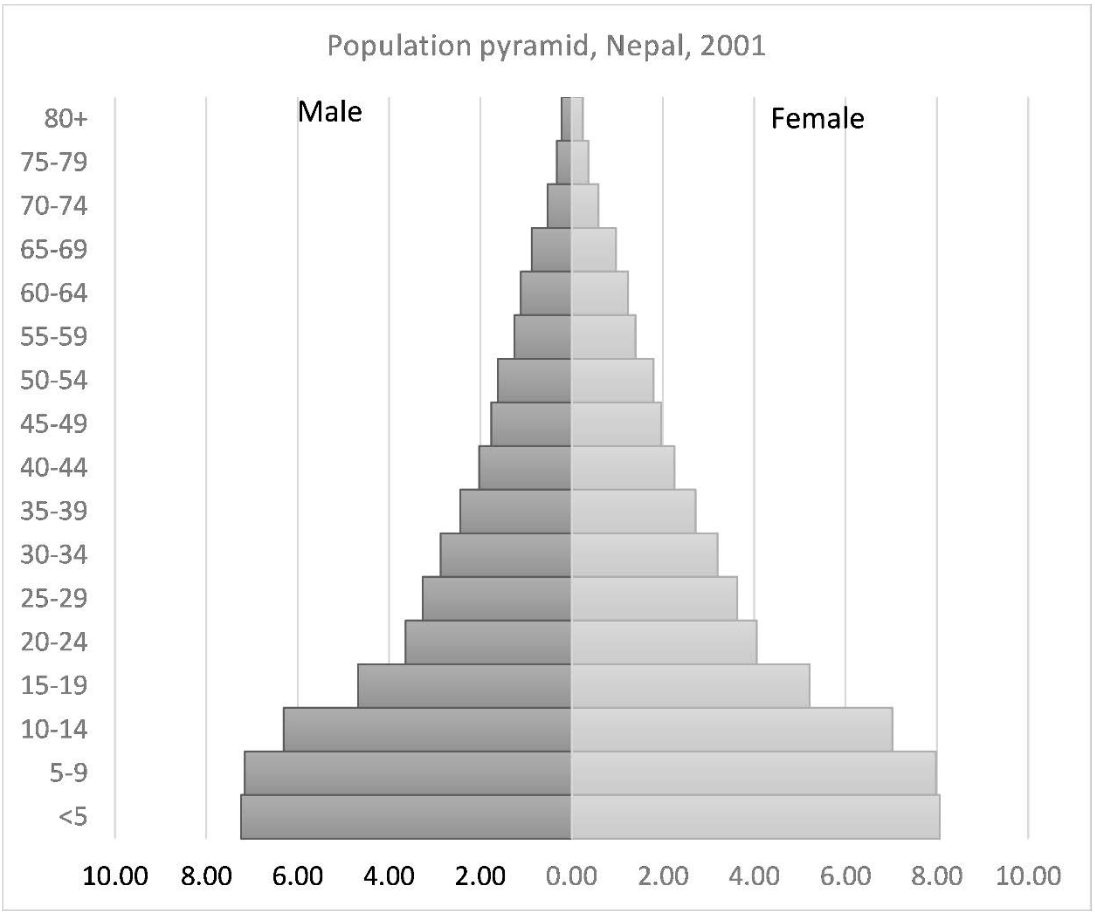

Supplementary Figure 2c. *Population pyramid, Nepal DHS, 2001. Population pyramids follow methodology as described in the manuscript (Methods section).*

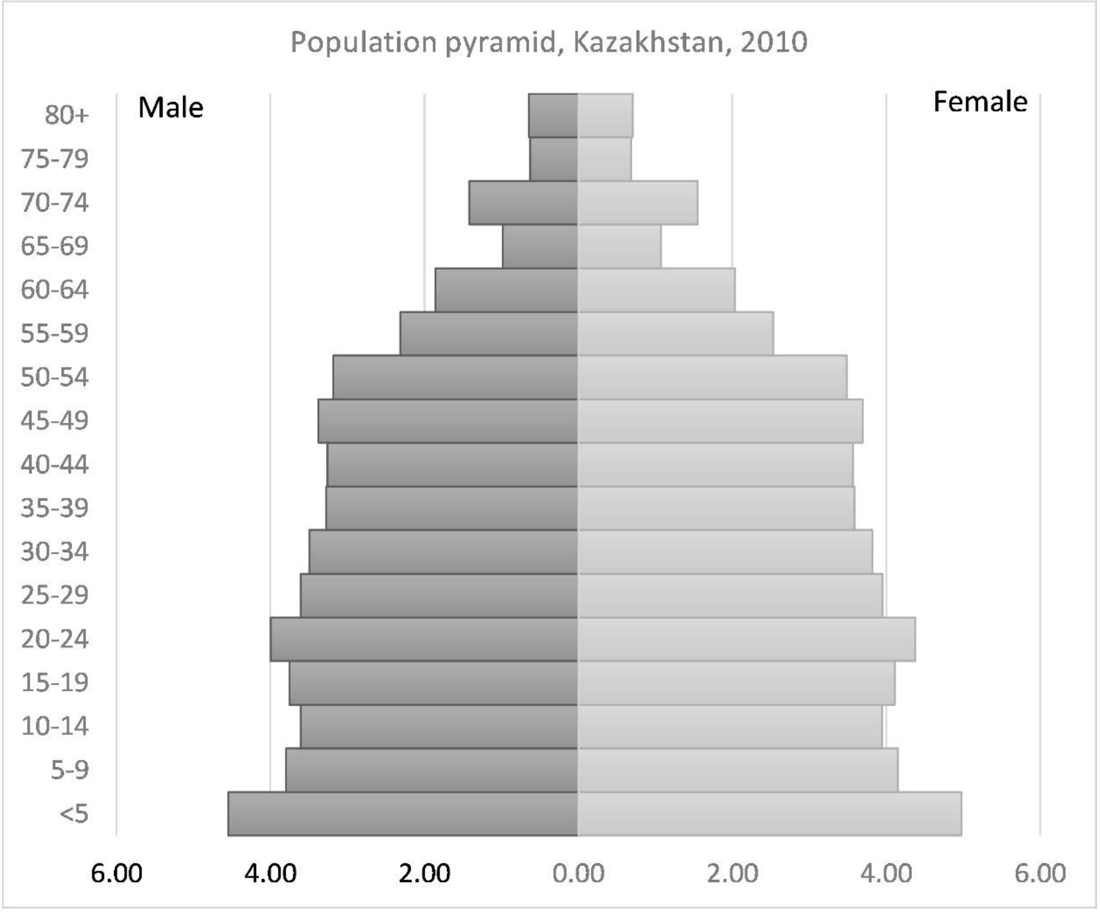

Supplementary Figure 3a. *Population pyramid, Kazakhstan MICS, 2010. Population pyramids follow methodology as described in the manuscript (Methods section).*

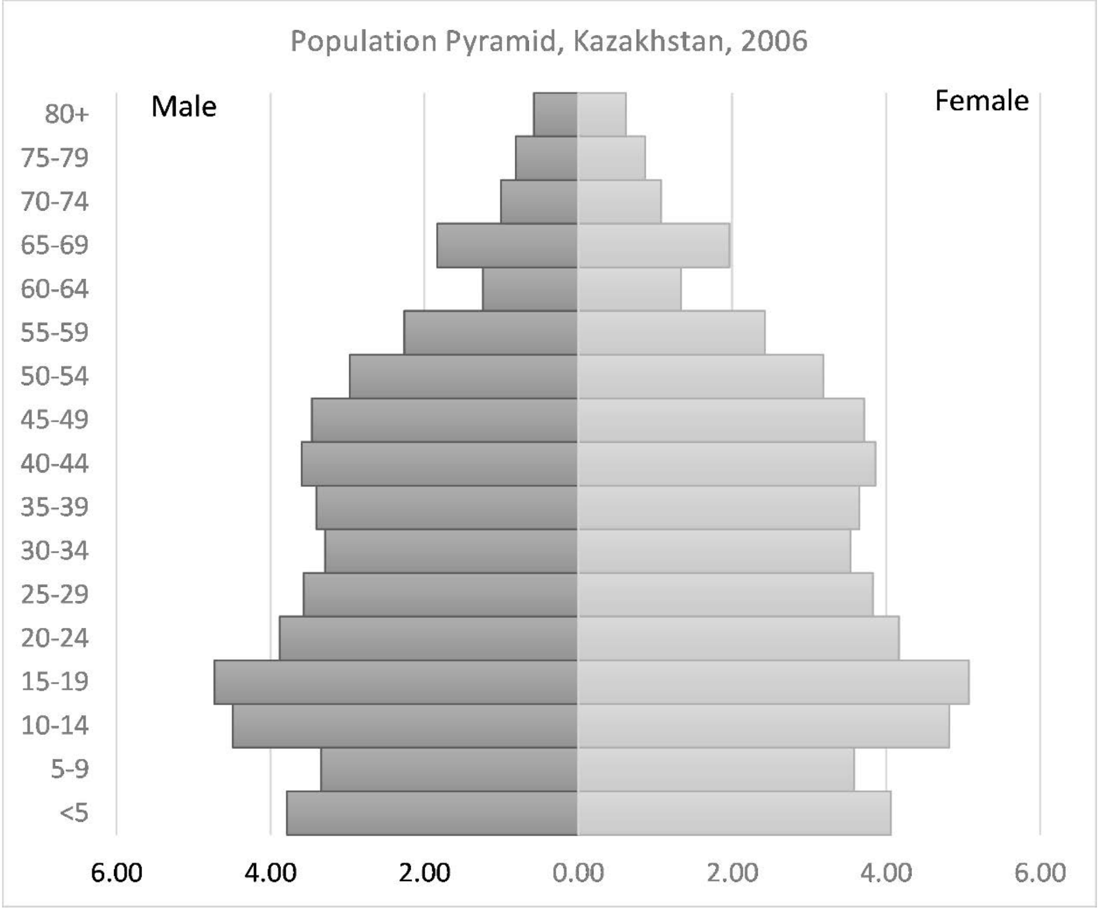

Supplementary Figure 3b. *Population pyramid, Kazakhstan MICS, 2006. Population pyramids follow methodology as described in the manuscript (Methods section).*

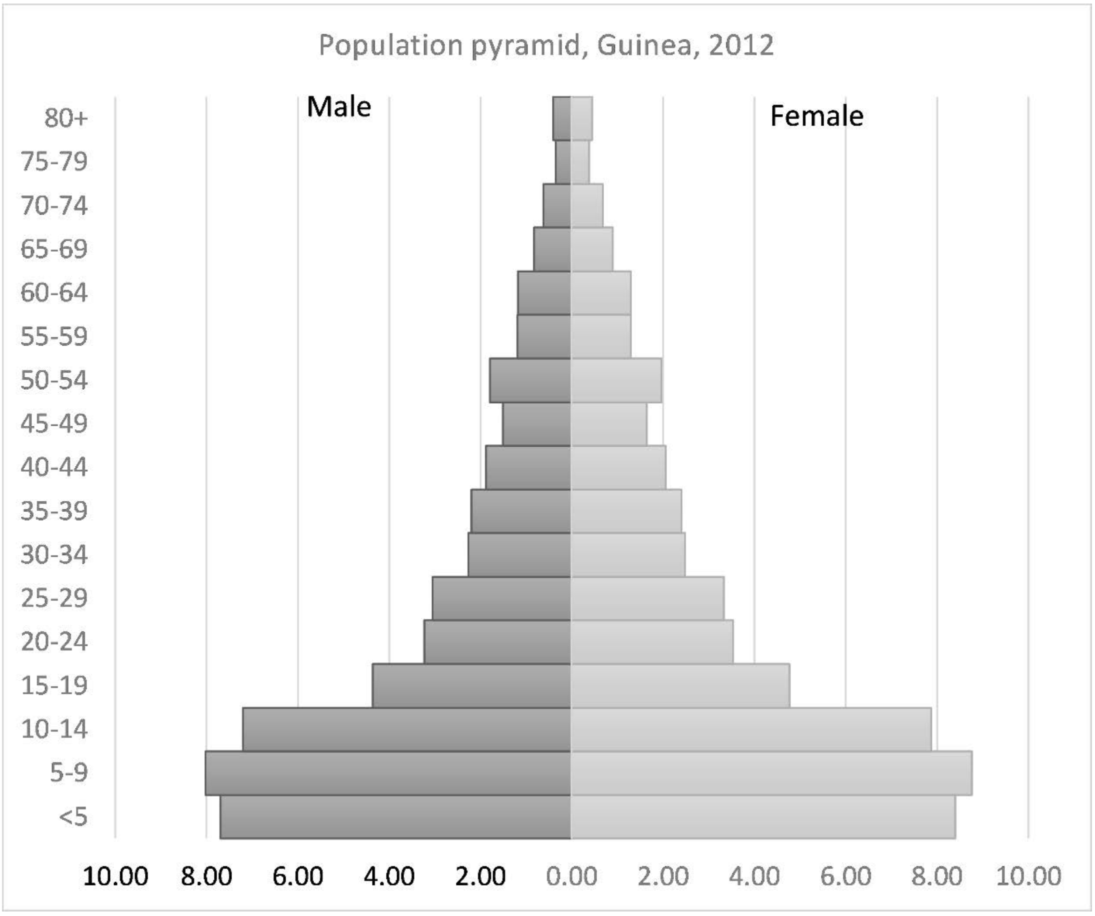

Supplementary Figure 4a. *Population pyramid, Guinea DHS, 2012. Population pyramids follow methodology as described in the manuscript (Methods section).*

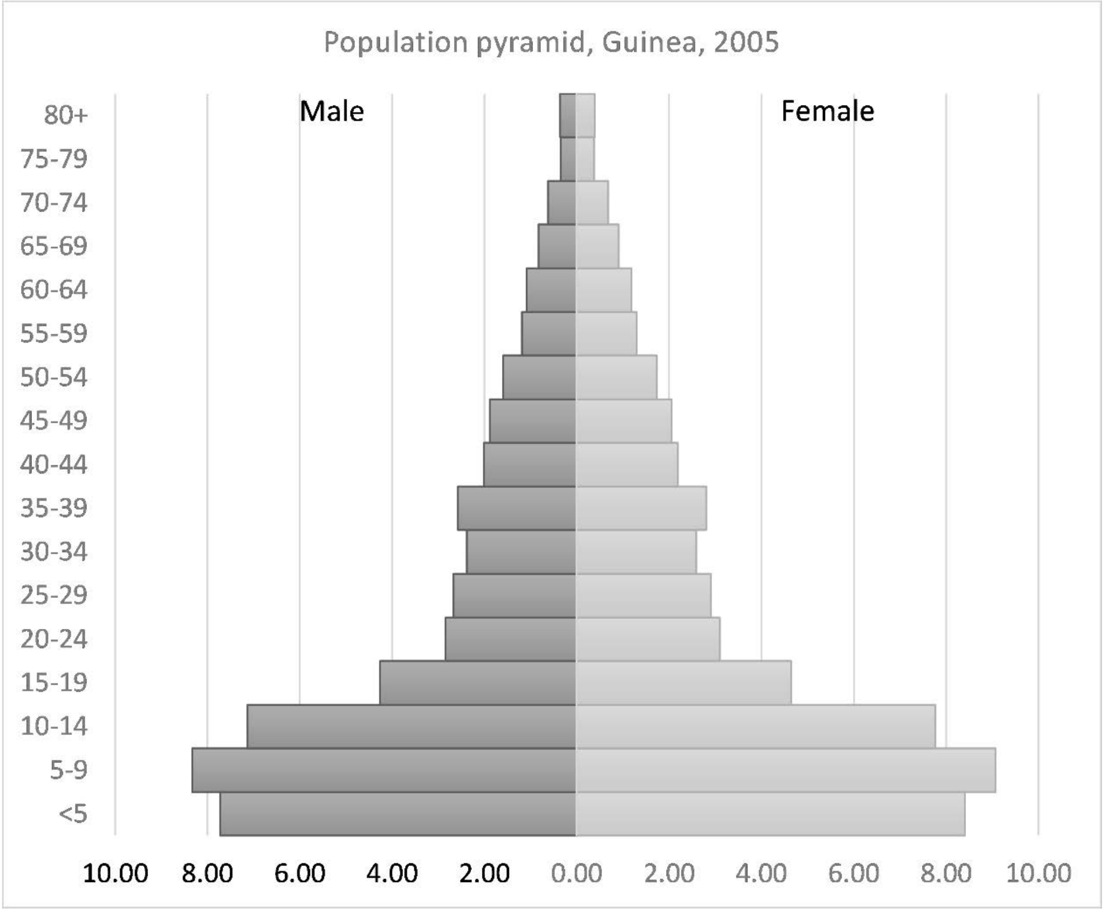

Supplementary Figure 4b. *Population pyramid, Guinea DHS, 2005. Population pyramids follow methodology as described in the manuscript (Methods section).*

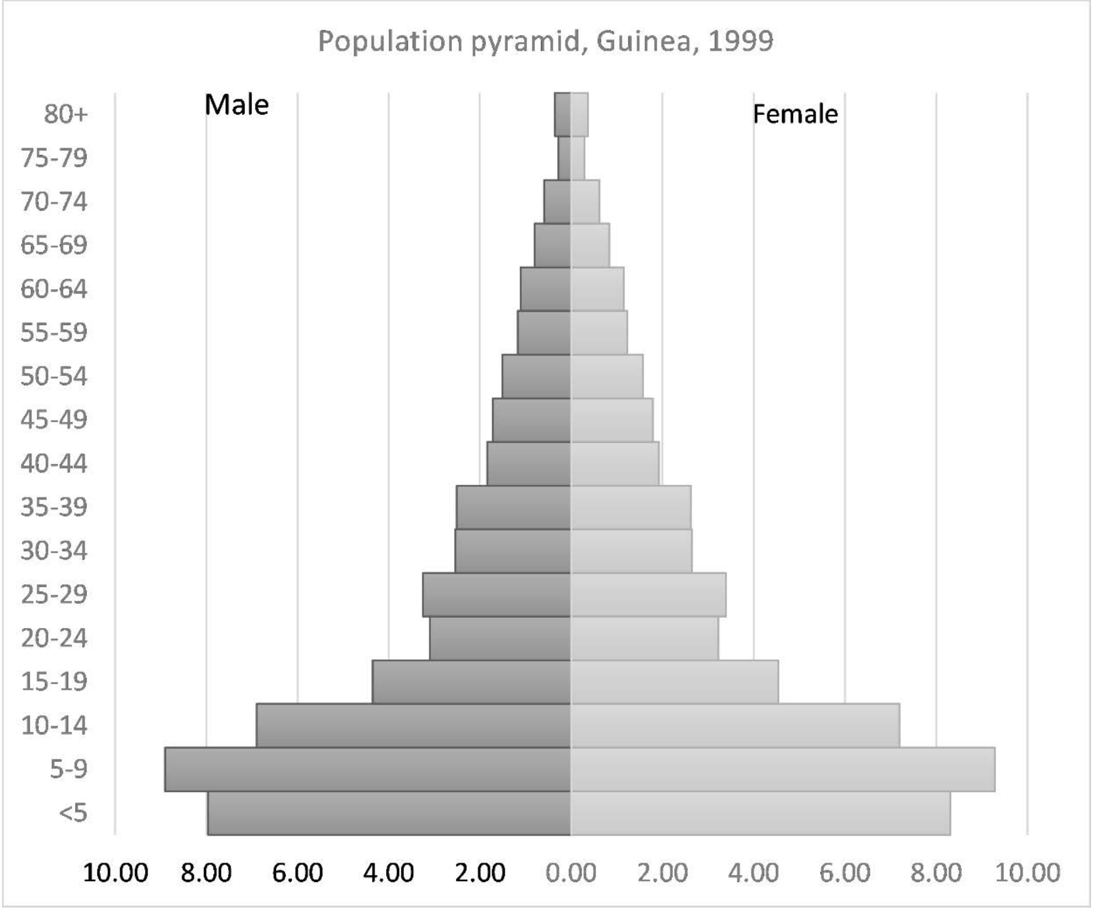

Supplementary Figure 4c. *Population pyramid, Guinea DHS, 1999. Population pyramids follow methodology as described in the manuscript (Methods section).*
